# Sodium sulfite (SoS) as decontamination strategy for *Fusarium*-toxin contaminated maize and its impact on immunological traits in pigs challenged with lipopolysaccharide (LPS)

**DOI:** 10.1007/s12550-020-00403-x

**Published:** 2020-09-09

**Authors:** Anh-Tuan Tran, Jeannette Kluess, Susanne Kersten, Andreas Berk, Marleen Paulick, Dian Schatzmayr, Sven Dänicke, Jana Frahm

**Affiliations:** 1 Institute of Animal Nutrition, Friedrich-Loeffler-Institut (FLI), Federal Research Institute for Animal Health, Braunschweig, Germany; 2 BIOMIN Holding GmbH, BIOMIN Research Center, Tulln, Austria

**Keywords:** Sodium sulfite, Deoxynivalenol, Immune cells, Lipopolysaccharide, Piglet

## Abstract

The main objective of this study was to evaluate the effects of sodium sulfite (SoS) treatment of maize and its impact on the porcine immune system in the presence of an LPS-induced systemic inflammation. Control maize (CON) and *Fusarium*-toxin contaminated maize (FUS) were wet-preserved (20% moisture) for 79 days with (+) or without (−) SoS and then included at 10% in a diet, resulting in four experimental groups: CON−, CON+, FUS−, and FUS+ with deoxynivalenol (DON) concentrations of 0.09, 0.05, 5.36, and 0.83 mg DON/kg feed, respectively. After 42-day feeding trial (weaned barrows, *n* = 20/group), ten pigs per group were challenged intraperitoneally with either 7.5 μg LPS/kg BW or placebo (0.9% NaCl), observed for 2 h, and then sacrificed. Blood, mesenteric lymph nodes, and spleen were collected for phenotyping of different T cell subsets, B cells, and monocytes. Phagocytic activity and intracellular formation of reactive oxygen species (ROS) were analyzed in both polymorphonuclear cells (PMN) and peripheral blood mononuclear cells (PBMC) using flow cytometry. Our results revealed that the impact of DON was more notable on CD3^+^CD4^+^CD8^+^ T cells in lymphoid tissues rather than in blood T cells. In contrast, SoS treatment of maize altered leukocyte subpopulations in blood, e.g., reduced the percentage and fluorescence signal of CD8^high^ T cells. Interestingly, SoS treatment reduced the amount of free radicals in basal ROS-producing PMNs only in LPS-challenged animals, suggesting a decrease in basal cellular ROS production (*p*_SoS*LPS_ = 0.022).

**Electronic supplementary material:**

The online version of this article (10.1007/s12550-020-00403-x) contains supplementary material, which is available to authorized users.

## Introduction

Deoxynivalenol (DON) belongs to the B-trichothecene mycotoxins produced from *Fusarium* species. Among the farm animals, pigs are known as the most susceptible species to DON exposure, responding with a reduction in feed intake and lower weight gain, resulting in economic losses for the farmer (EFSA 2004). In the temperate climate regions, DON often occurs in cereal grains, especially wheat and maize (EFSA 2013). Moreover, DON contamination in the field cannot completely be avoided due to weather conditions. Additionally, DON is only negligibly degraded by feed processing and therefore decontamination methods are required to inactivate DON before cereal grains are used for the production of complete feeds (Awad et al. 2010). Although various decontamination strategies were investigated, an effective procedure is still needed (Kabak et al. 2006; He et al. 2010). Moreover, easy-to-use inactivation procedures are required at farm level where cereals are directly used for feeding. The simple wet preservation of *Fusarium*-toxin contaminated cereal grains with sodium sulfite (Na_2_SO_3_, SoS) or sodium metabisulfite (Na_2_S_2_O_5_, SBS), using a defined moisture content and acidification with propionic acid, has shown to decrease DON concentration via the formation of sulfonated derivatives of DON, the so-called DON sulfonates (DONS) (Young et al. 1987; Schwartz et al. 2013; Schwartz-Zimmermann et al. 2014; ). The SoS- and SBS-induced decrease of DON concentration in cereal grains was reflected by a concomitant detection of low DON concentrations in blood and other physiological specimen (Dänicke et al. 2005; Dänicke et al. 2008; Paulick et al. 2018; Tran et al. 2018a) as well as an improved performance comparable with that observed in control groups fed non-contaminated diets (Paulick et al. 2018).

Despite positive effects on the piglet performance, several non-specific effects of SBS treatment were recorded when pigs were fed SBS-treated diets irrespective of DON contamination (Dänicke et al. 2008; Dänicke et al. 2012), such as a plasma protein increase, stimulated liver function as determined by the ^13^C-methacetin breath test, and an increased stimulation capacity of peripheral blood mononuclear cells (PBMC). The liver, besides its function as a main metabolic organ, also acts as a secondary immunological organ, which initiates and mediates the acute phase response as an innate immune mechanism (Crispe 2009); therefore, a consequential interference of SBS treatment with the immune system remains to be elucidated.

Lipopolysaccharides (LPS) are a major component of the cell wall of Gram-negative bacteria (Palsson-McDermott and O’Neill 2004) and are known as pathogen-associated molecular patterns (PAMPs), capable of inducing an acute phase response. The latter consists of immediate effects such as releasing soluble mediators like tumor necrosis factor-alpha (TNF-α) and interferon-β (IFN-β) from immune cells into the circulation as well as secondary inflammatory effects, for example, an increased aspartate aminotransferase (AST) activity and bilirubin concentration leading to tissue and cellular injuries (Dänicke et al. 2013; Kuzmich et al. 2017). Due to these characteristics, LPS is experimentally often used as a defined immune challenge in animal models investigating the responsiveness of the immune system (Wyns et al. 2015).

Applying this LPS model, we could show recently that both, SoS treatment of maize and LPS-induced systemic inflammation, altered the differential white blood cell counts (total leukocytes, lymphocytes, granulocytes, monocytes) of piglets in an interactive manner (Tran et al. 2018b). However, based on this general evaluation of the white blood count, no information about possible mechanisms of these global changes could be gathered. Thus, lymphocytes were further phenotyped investigating the effects of SoS wet-preservation on lymphocyte subpopulations, such as T helper and cytotoxic T cells. As granulocytes and monocytes directly or indirectly influence the number and function of lymphocytes and other immune cells, we further examined their main functions such as phagocytic activity and capability to mount an oxidative burst.

## Material and methods

### Animal experiment

The experiment was performed at the experimental station of the Institute of Animal Nutrition, Friedrich-Loeffler-Institute (FLI), Brunswick, Germany, in compliance with the European Community regulations concerning the protection of experimental animals and was approved by the Lower Saxony State Office for Consumer Protection and Food Safety (LAVES), Germany (file number: 33.92-42502-04-13/1153).

All samples were derived from a weaned piglet trial, set up in a 2 × 2 × 2 factorial design (maize batch × sodium sulfite × LPS challenge) comprising an acute LPS challenge at the end of a 42-day feeding period (Tran et al. 2018b). Briefly, two maize batches were produced, a control batch (CON) with a background contamination of the two major *Fusarium* mycotoxins deoxynivalenol (DON) and zearalenone (ZEN) and a batch experimentally inoculated with *Fusarium* spores (FUS), containing high levels of DON and ZEN (Paulick et al. 2015). Each batch of maize kernels was subdivided and wet-preserved (20% moisture, 15 g propionic acid/kg maize), either with or without (**+/−**) the addition of 5 g SoS/kg maize (Na_2_SO_3_: CAS-no. 7757-83-7, ≥ 98%, p.a., ACS, water free; Carl Roth GmbH & Co KG, Karlsruhe/Germany) for 79 days. After preservation, the resulting four maize batches were ground and blended into experimental diets containing 35% barley, 27.3% wheat, 10% maize, 23% soybean meal, 1.5% soya bean oil, 1% vitamin and mineral premix, 2.2% synthetic amino acids (HCl-lysine, DL-methionine, L-threonine, L-tryptophan), and a commercial phytase. This resulted in four experimental diets: CON− (control maize, without SoS; 0.09 mg DON/kg feed, ZEN < LOD), CON+ (control maize, with SoS; 0.05 mg DON/kg feed, ZEN < LOD), FUS− (contaminated maize, without SoS; 5.36 mg DON/kg feed, 0.29 mg ZEN/kg feed), and FUS+ (contaminated maize, with SoS; 0.83 mg DON/kg feed, 0.27 mg ZEN/kg feed).

Eighty male castrated crossbred piglets (7.59 ± 0.92 kg) were allotted equally—based on body weight—to the four experimental groups, group-housed (4 piglets/pen; 20 pigs/group = 5 pens/group), and offered feed and water for *ad libitum* consumption. After 42 days, ten pigs of each dietary group were allocated to the challenge trial, while the remaining pigs (*n* = 10/diet) were slaughtered for further aspects (Tran et al. 2018a). Detailed procedures of the LPS challenge were described in Tran et al. (2018b). Briefly, these piglets were injected intraperitoneally either with 7.5 μg LPS/kg BW (*Escherichia coli* serotype O111:B4, Sigma-Aldrich, Steinheim, Germany, L 2630; CON−/LPS, CON+/LPS, FUS−/LPS, FUS+/LPS; *n* = 5 per treatment group) or with 0.9% NaCl as placebo (volume ~ 6.5 mL/animal; CON−/NaCl, CON+/NaCl, FUS−/NaCl, FUS+/NaCl; *n* = 5 per treatment group). Two hours after the challenge, animals were electrically stunned, blood samples taken immediately from neck vessels for further analyses, and pigs then sacrificed by exsanguination. Pigs were immediately dissected, tissue samples of mesenteric lymph nodes and spleen collected and directly placed into a cardioplegia solution (Custodiol, Dr. Franz Köhler Chemie GmbH, Bensheim, Germany) until cell preparation procedures.

### Analytical methods

#### Cell preparations

Cells from mesenteric lymph nodes were isolated by a modified method according to Solano-Aguilar et al. (2000). Briefly, serosa and surrounding tissues from mesenteric lymph nodes were removed. Then, mesenteric lymph nodes were transferred into a culture dish with cold PBS (phosphate-buffered saline), cut medial and longitudinal with a scalpel to obtain a cell suspension. The suspension was filtered with a sieve kept on ice and centrifuged at 300x*g* at 4 °C for 10 min. Next, the supernatant was removed and the pellet was resuspended in HEPES-buffered saline (HBS). After one filtration, this cell suspension was used for staining with antibodies for flow cytometry.

Isolation of splenic cells was performed according to Renner et al. (2012). In brief, spleen tissue was minced with a scalpel, placed into sterile HEPES-buffered solution (HBS), and smoothed through a cell strainer into Petri dishes for erythrocytes’ lysis. After centrifugation (250×*g*, 5 min) and washing in HBS, cells were resuspended in RPMI 1640 medium, supplemented with fetal bovine serum, penicillin-streptomycin, mercapto-ethanol, and L-glutamine (Biochrom AG, Berlin, Germany). Subsequently, these cells were stained with antibodies for flow cytometric analysis.

#### Phenotyping of leukocyte subsets

For phenotyping of leukocyte subsets, single cell suspensions (1 × 10^6^ cells/mL, in duplicate/sample) of cells from either mesenteric lymph node or splenocytes or 50 μl EDTA whole blood were incubated with monoclonal antibodies (Table 1) as double stain (monocytes, B cells) or triple stain (T cells) for 30 min on ice in the dark. After washing, the cell suspensions from tissue samples were resuspended in HBS and analyzed with a FACSCanto^TM^ II flow cytometer (BD Biosciences, San Jose, CA, USA). The whole blood samples were further processed by incubating for 10 min with lysis buffer (BD Pharm Lyse, BD Biosciences, San Jose, CA, USA), centrifugation (5 min, 250×*g*, 4 °C), and resuspension in HBS.

**Table 1:** List of monoclonal antibodies (mAb) as applied in flow cytometric analysis of leukocytes (FACS™ II Canto flow cytometer, BD Biosciences, San Jose, CA, USA)

Specificity	mAb	Fluorescent dye	Manufacturer
CD3	Mouse Anti-Pig CD3ε	Alexa Fluor®647	BD Bioscience, San Jose, USA
CD4	Mouse Anti-Pig CD4	FITC	AbD, seroTEC, Oxford, UK
CD8	Mouse Anti-Pig wCD8	RPE	AbD, seroTEC, Oxford, UK
CD21	Mouse Anti-Human CD21	PE	BD Biosciences, San Jose, USA
CD14	Mouse Anti-Pig CD14	FITC	AbD, seroTEC, Oxford, UK
Isotype control	mouse IgG1	Alexa Fluor®647	AbD, seroTEC, Oxford, UK
Isotype control	mouse IgG2a	RPE	AbD, seroTEC, Oxford, UK
Isotype control	mouse IgG2b	FITC	AbD, seroTEC, Oxford, UK

For measurement, an acquisition gate was set for blood peripheral mononuclear cells (PBMC = lymphocytes, monocytes) according to its side scatter and forward scatter characteristics for each tissue (blood, spleen, mesenteric lymph node). A minimum of 10,000 cells was evaluated with FACSDiva^TM^ software 6.1.3 (BD Biosciences, San Jose, CA, USA). The gating strategies for T cells (S1) and B cells (S2) are detailed in supplementary material.

B cells (CD21^+^) and monocytes (CD14^+^) were characterized by the expression of their respective epitope. Four T cell subsets were defined as follows: CD3^+^CD4^+^CD8^−^ (T helper cells), CD3^+^CD4^−^CD8^+^ (cytotoxic T cells), CD3^+^CD4^−^CD8^−^ (undifferentiated T cells), and CD3^+^CD4^+^CD8^+^ (double-positive T cells). The expression density of each CD epitope per cell was characterized by their mean fluorescence intensity (MFI). The total CD3^+^CD4^-^CD8^+^ cells were further separated in two subsets according to their fluorescence intensity classifying cells of low (CD8^low^) or high expression (CD8^high^) of the CD8 marker. Furthermore, the ratio between CD3^+^CD4^+^CD8^−^ and CD3^+^CD4^−^CD8^+^ cells (CD4^+^/CD8^+^) was calculated.

#### Intracellular production of reactive oxygen species

Dihydrorhodamine 123 (DHR, Molecular Probes, Eugene, Oregon, USA) is a non-fluorescent molecule capable of passing the cell membrane and can be oxidized to its fluorescent product rhodamine 123 (R123^+^) by ROS. Thus, DHR is routinely used to measure intracellular ROS generation by flow cytometry. The capacity of ROS production in polymorphonuclear cells (PMN = granulocytes) and PBMC was determined using a FACSCanto^TM^ II flow cytometer (BD Biosciences, San Jose, CA, USA). Fifty microliters of whole blood samples (in duplicates) were incubated for 15 min at 37 °C with 40 mM DHR alone (basal production) or with 20 nM tetradecanoyl-12,13-phorbol acetate (TPA, Sigma Aldrich, Taufkirchen, Germany) in order to stimulate an oxidative burst. After 10 min lysis of erythrocytes (BD Pharm Lyse, BD Biosciences, San Jose, CA, USA), cells were washed with HBS and the formation of R123^+^ was measured. At least 10,000 cells of each population were analyzed selecting the appropriate gates. Percentage of ROS-producing PMN and PMBC and the intracellular level of ROS production (MFI) were determined.

#### Phagocytosis assay

The phagocytic activity of PMN and PBMC was assessed by a commercial kit, Phagotest^TM^ (Glycotope Biotechnology, Heidelberg, Germany) according to the manufacturer’s instructions. Briefly, 100 μl heparinized whole blood (in duplicates) was incubated with FITC-labelled *E. coli* bacteria for either 10 min at 37 °C or 10 min in an ice bath for negative control. After quenching and lysis of erythrocytes, cells were washed and propidium iodide (PI) was added to stain the DNA. Fluorescence intensity was measured using FACSCanto^TM^ II (BD Biosciences, San Jose, CA, USA). PMN and PBMC cells were gated according to their size and granularity based on measurements of forward and size scatter (S1). At least 10,000 cells of each population were analyzed with FACSDiva^TM^ software 6.1.3 (BD Biosciences, San Jose, CA, USA). Percentage of phagocyting PMN and PMBC as well as the phagocytic capacity per cell was characterized by their MFI.

### Statistics

Data were statistically analyzed as a 2 × 2 × 2 factorial design, using PROC MIXED (SAS Institute 2004), with maize batch (control maize vs. *Fusarium-*toxin contaminated maize [FUS]), SoS treatment (with vs. without Na_2_SO_3_ [SoS]), and LPS challenge (NaCl vs. LPS [LPS]) as well as all their interactions (FUS*SoS, FUS*LPS, SoS*LPS, FUS*SoS*LPS) as fixed factors. Differences were deemed significant at *p* < 0.05 and Student’s *t* test was applied as *post hoc* procedure. Results are presented as least square means (LSmeans) and pooled standard error of the mean (PSEM).

## Results

### T cell subsets in peripheral blood and lymphatic organs

**Fig. 1:**
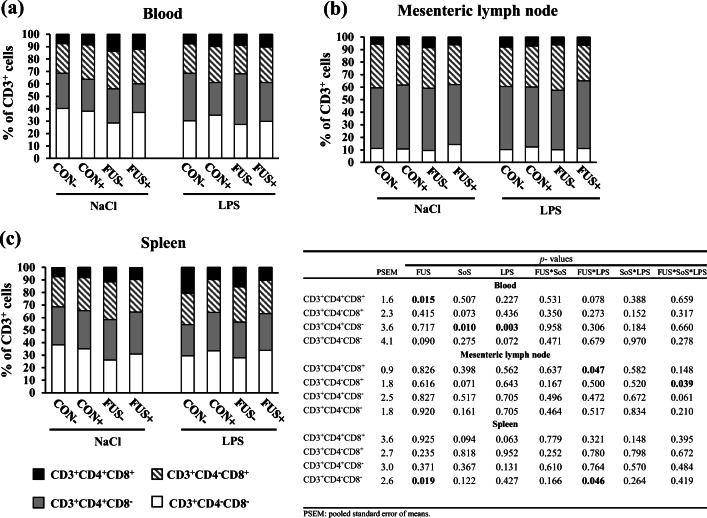
Distribution pattern of four T cell subsets (% of CD3^+^ cells) in blood (**a**), mesenteric lymph node (**b**), and spleen (**c**) in piglets receiving experimental diets for 5 weeks and subjected to a subsequent acute LPS challenge. Diets contained control (CON) or *Fusarium-*toxin contaminated maize (FUS), wet-conserved with or without 5 g SoS/kg maize sodium sulfite (+/−) and piglets were injected with 7.5 μg LPS/kg BW or 0.9% NaCl. Data represent LSmeans (*n* = 5) for each T cell subset

T cells in peripheral blood and lymphatic tissues were characterized by their expression of the CD3 protein complex and further differentiated in four subsets according to the co-expression of CD4 and CD8 epitopes. The distribution of T cell phenotypes as percentage of all CD3^+^ cells in blood, mesenteric lymph node, and spleen, including their respective *p* values, are presented in Fig. 1.

In blood (Fig. 1a), we observed a significant impact of the factor FUS (*p*_FUS_ = 0.015), with an increase in double-positive cells (CD3^+^CD4^+^CD8^+^) in pigs receiving FUS-contaminated diets (8.4 vs. 11.2%). Furthermore, CD4^+^ cells (CD3^+^CD4^+^CD8^−^) were decreased by SoS treatment (*p*_SoS_ = 0.010, 33.7 vs. 26.6%) but increased in LPS-challenged pigs (*p*_LPS_ = 0.003, 26.0 vs. 34.2%). No interactions were evident in blood T cell subsets.

In mesenteric lymph node (Fig. 1b), a significant interaction between FUS and LPS challenge (*p*_FUS*LPS_ = 0.047) was observed for the double-positive cells (CD3^+^CD4^+^CD8^+^) due to a decline in the pooled group FUS/NaCl while the other three groups remained unaltered. Moreover, CD8^+^ cells (CD3^+^CD4^−^CD8^+^) significantly displayed an interaction between FUS, SoS treatment, and LPS challenge (*p*_FUS*SoS*LPS_ = 0.039) due to a clear increase in group FUS−/LPS and a strong reduction in group FUS+/LPS while the other groups remained unchanged.

In spleen (Fig. 1c), the percentage of double-negative cells (CD3^+^CD4^−^CD8^−^) was lower in pooled groups FUS/NaCl, CON/LPS, and FUS/LPS compared with group CON/NaCl, which resulted in a significant interaction between the main factors FUS and LPS challenge (*p*_FUS*LPS_ = 0.046).

The intensity of the fluorescence signal as an indicator of cellular activity remained unaffected in total CD8^+^ T cells (S3a) in lymph node (4.5 ± 0.3 × 10^3^) and spleen (3.1 ± 0.8 × 10^3^). However, in blood, statistical analysis revealed a significant increase of MFI in CD8^+^ cells due to FUS feeding (*p*_FUS_ = 0.008). Factor SoS showed a contrasting effect (p_SoS_ = 0.001) with a decrease in MFI levels due to SoS treatment. In CD4^+^ cells, the MFI (S3b) was unaltered by any treatment with 2.0 ± 0.1 × 10^3^ in blood, 2.4 ± 0.1 × 10^3^ in lymph node, and 2.6 ± 0.1 × 10^3^ in spleen.

**Fig. 2:**
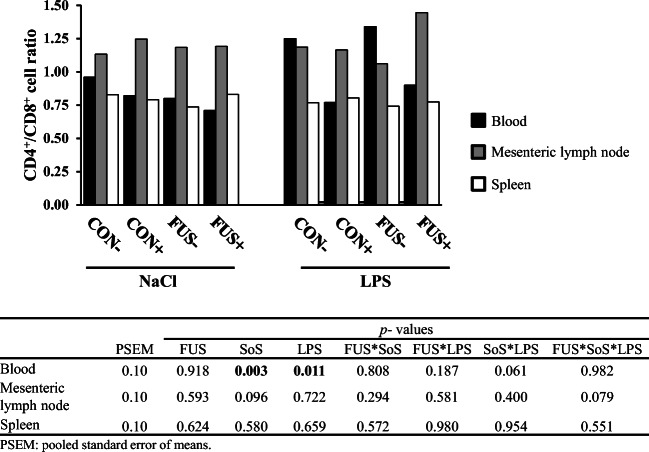
Relationship between T helper and cytotoxic T cells (CD4^+^ to CD8^+^ ratio) in blood, mesenteric lymph node, and spleen (LSMeans, *n* = 5) in piglets receiving experimental diets for 5 weeks and subjected to a subsequent acute LPS challenge. Diets contained control (CON) or *Fusarium*-toxin contaminated maize (FUS), wet-conserved with or without 5 g SoS/kg maize sodium sulfite (+/−) and piglets were injected with 7.5 μg LPS/kg BW or 0.9% NaCl

Although some alterations of T cell subpopulations were found in the specimen analyzed, the CD4^+^/CD8^+^ ratio (Fig. 2) was only significantly affected by treatments in blood samples rather than spleen and mesenteric lymph node: SoS treatment (*p*_SoS_ = 0.003) decreased the CD4^+^/CD8^+^ ratio due to a decrease in the CD4^+^ cells, whereas LPS (*p*_LPS_ = 0.011) increased the ratio, likely due to the increase in CD4^+^ and constancy of CD8^+^ cells. There was no interaction between the main factors. Furthermore, CD4^+^/CD8^+^ ratio differed significantly due to site of cell residence: the highest CD4^+^/CD8^+^ ratio was observed in the mesenteric lymph node, whereas the CD4^+^/CD8^+^ ratio in blood and spleen appeared comparable (*p*_Localization_ < 0.001).

**Fig. 3:**
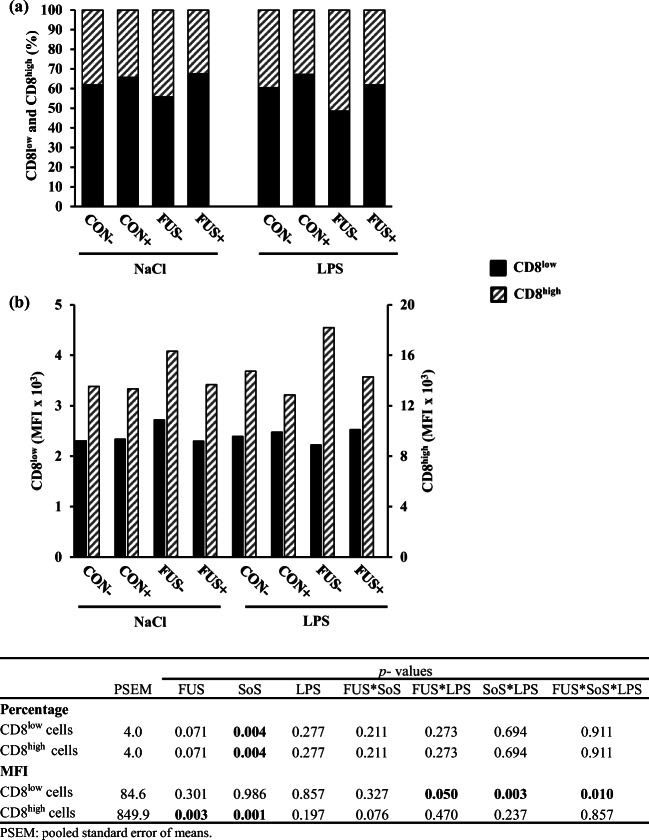
Proportion of cytotoxic (**a**) CD8^low^ and CD8^high^ T cells in blood (LSMeans, *n* = 5) and their respective mean fluorescence intensity (MFI; **b**) in piglets receiving experimental diets for 5 weeks and subjected to a subsequent acute LPS challenge. Diets contained control (CON) or *Fusarium*-toxin contaminated maize (FUS), wet-conserved with or without 5 g SoS/kg maize sodium sulfite (+/−) and piglets were injected with 7.5 μg LPS/kg BW or 0.9% NaCl

In pigs, CD8^+^ T cells are unique as they show two subpopulations with high and with low expression (CD8^low^ and CD8^high^) of the CD8 subsets (Lunney and Pescovitz 1987; Gerner et al. 2009), denoting differences in cellular function. These subpopulations and their respective MFI in peripheral blood are displayed in Fig. 3. The main factor SoS treatment showed a significant effect on the percentage of both CD8^low^ and CD8^high^ cells: CD8^low^ cells were increased whereas CD8^high^ cells were conversely decreased (Fig. 3a). A significant interaction between FUS, SoS treatment, and LPS challenge was found for the MFI (Fig. 3b) of CD8^low^ cells due to an increase in group FUS−/NaCl and a drop in group FUS−/LPS, whereas the other groups remained unaltered. Changes in MFI of CD8^high^ cells were also observed: MFI was increased in pigs receiving FUS-contaminated diets (13.6 × 10^3^ vs. 15.6 × 10^3^), whereas a depressing effect of SoS treatment was shown (15.7 × 10^3^ vs. 13.5 × 10^3^). In mesenteric lymph node, there were no alterations in percentage and MFI of CD8^low^ (67.4 ± 5.8%, 2.7 ± 0.2 × 10^3^) and CD8^high^ cells (32.5 ± 5.8%, 8.1 ± 0.4 × 10^3^). Similarly, the percentage and MFI of both CD8^low^ and CD8^high^ cells in spleen remained unaltered (CD8^low^: 86.3 ± 3.6%, 2.0 ± 0.1 × 10^3^; CD8^high^: 13.5 ± 3.5%, 9.3 ± 1.0 × 10^3^).

### B cell and monocytes in blood and lymphatic organs

**Fig. 4:**
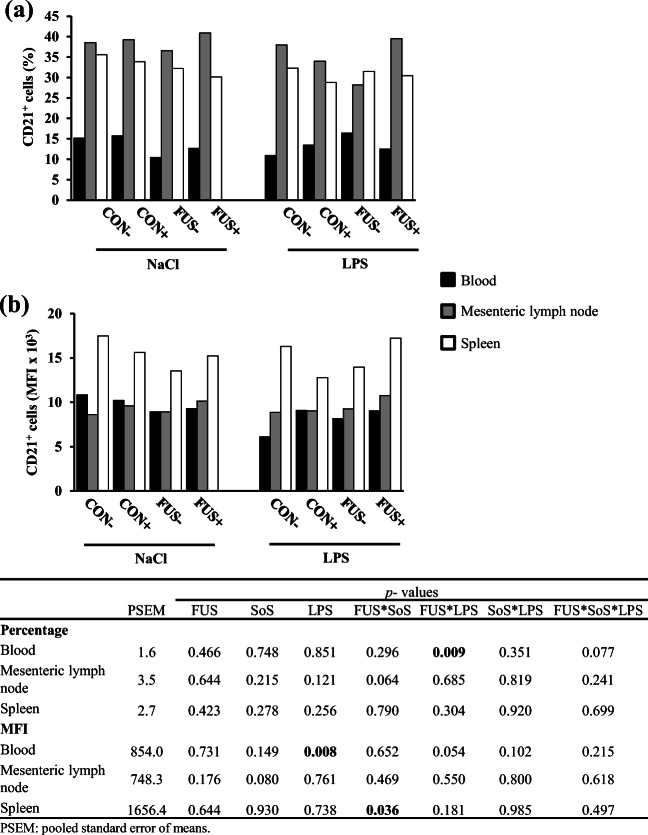
Distribution of CD21^+^ B cells (**a**) in blood, mesenteric lymph node, and spleen (LSMeans, *n* = 5) and their respective mean fluorescence intensity (MFI; **b**) in piglets receiving experimental diets for 5 weeks and subjected to a subsequent acute LPS challenge. Diets contained control (CON) or *Fusarium*-toxin contaminated maize (FUS), wet-conserved with or without 5 g SoS/kg maize sodium sulfite (+/−) and piglets were injected with 7.5 μg LPS/kg BW or 0.9% NaCl

B cells were characterized by the expression of CD21^+^ epitope. The percentage of CD21^+^ cells was altered in blood only (Fig. 4a), with a significant interaction between FUS and LPS: pooled groups FUS/NaCl and CON/LPS were lower compared with their respective counterparts CON/NaCl and FUS/LPS. The MFI of CD21^+^ cells in blood was significantly reduced in LPS-challenged pigs compared with their placebo counterparts. In spleen, an interaction between FUS and SoS treatment was evident: in CON-fed groups, SoS treatment (CON+) decreased MFI, whereas in their FUS counterparts, the situation was vice versa. There were no changes in CD21^+^ cells in mesenteric lymph node, neither in percentage nor in MFI.

**Fig. 5:**
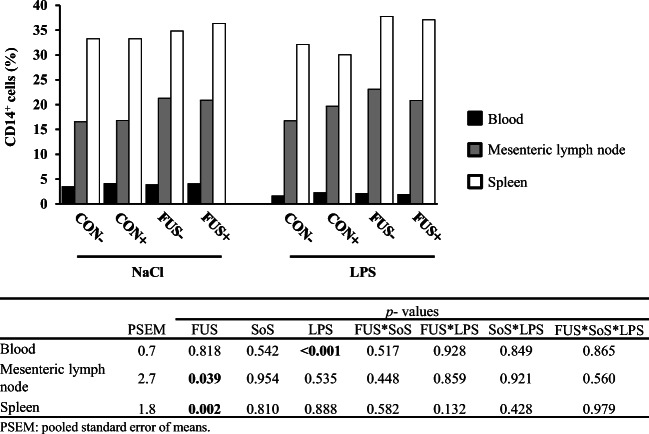
Proportion of CD14^+^ monocytes in blood, mesenteric lymph node, and spleen (LSMeans, *n* = 5) in piglets receiving experimental diets for 5 weeks and subjected to a subsequent acute LPS challenge. Diets contained control (CON) or *Fusarium-* toxin contaminated maize (FUS), wet-conserved with or without 5 g SoS/kg maize sodium sulfite (+/−) and piglets were injected with 7.5 μg LPS/kg BW or 0.9% NaCl

Monocytes, identified as CD14^+^ cells by flow cytometry, showed a marked difference between blood, mesenteric lymph node, and spleen (Fig. 5): < 5% in blood, ~ 20% in mesenteric lymph node, and ~ 30% in spleen. In blood, percentage of CD14^+^ cells was significantly decreased in LPS-challenged pigs (NaCl vs. LPS: 3.9 vs. 2.0%). In both lymphatic tissues, feeding FUS diets significantly increased the CD14^+^ proportion as compared with CON diets: in mesenteric lymph node from 17.5 to 21.5% and in spleen from 32.2 to 36.5%. There were no significant changes due to other factors or a significant interaction between factors. The MFI of CD14^+^ cells in blood, mesenteric lymph node, and spleen was not altered by any treatment and its values averaged at 1.5 ± 0.2 × 10^3^, 2.5 ± 0.3 × 10^3^, and 2.6 ± 0.2 × 10^3^, respectively.

### Intracellular production of reactive oxygen species

**Fig. 6:**
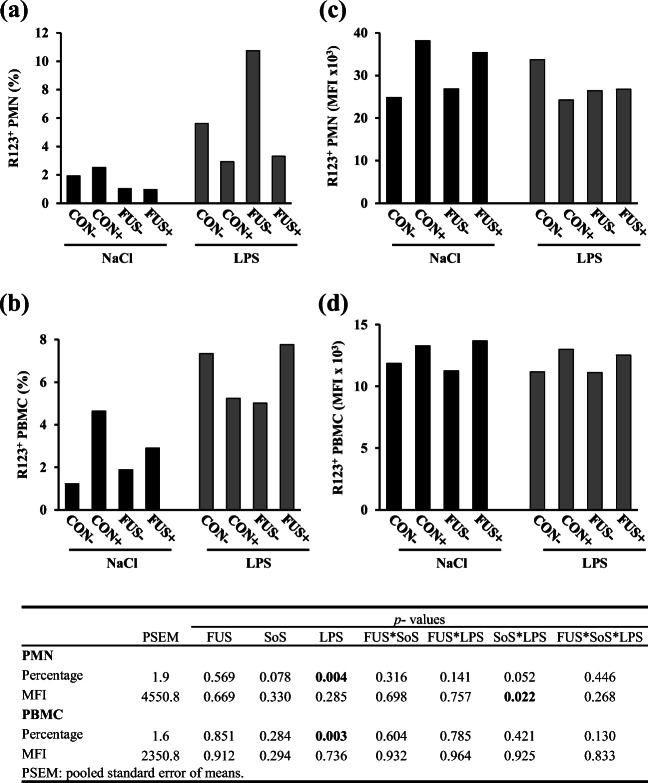
Intracellular basal (= non-stimulated) ROS production was evaluated in PMN (= granulocytes) and PBMC (incl. monocytes) with the fluochrome rhodamine R123 (R123^+^). The percentage of R123^+^ PMN (**a**) and PBMC (**b**) and their respective MFI values (**c**, **d**) are presented as LSMeans (*n* = 5). Piglets received experimental diets for 5 weeks and were subjected to a subsequent acute LPS challenge. Diets contained control (CON) or *Fusarium*-toxin contaminated maize (FUS), wet-conserved with or without 5 g SoS/kg maize sodium sulfite (+/−) and piglets were injected with 7.5 μg LPS/kg BW or 0.9% NaCl

The basal ROS production (=R123^+^ cells) was measured in PMN and PBMC and the percentage of positive cells as well as the MFI as functional marker is depicted in Fig. 6. Both the proportion of ROS-producing PMN (Fig. 6a) and PBMC (Fig. 6b) were significantly increased in LPS-challenged animals compared with their placebo counterparts. Moreover, the MFI in PMN (Fig. 6c) showed a significant interaction between SoS treatment and LPS challenge: whereas in NaCl groups, there was no marked impact of SoS-treatment, SoS-treatment resulted in a significant decrease in LPS-challenged groups. In PBMC, there was no impact on MFI (Fig. 6d). Both PMN and PBCM were stimulated with TPA in order to induce an oxidative burst and both cell populations showed a clear increase in percentage (PMN 97.8 ± 0.7%; PBMC 24.8 ± 5.4%) and MFI (PMN 80.3 ± 6.8 × 10^3^; PBMC 99.0 ± 21.3 × 10^3^) after stimulation. This indicates that cells were capable of oxidative burst and responded well to the stimulation. However, stimulating properties and capacity of radical production in TPA-stimulated cells remained unaffected by any treatment in PMN and PBMC.

### Phagocytosis of PMN and PBMC

**Fig. 7:**
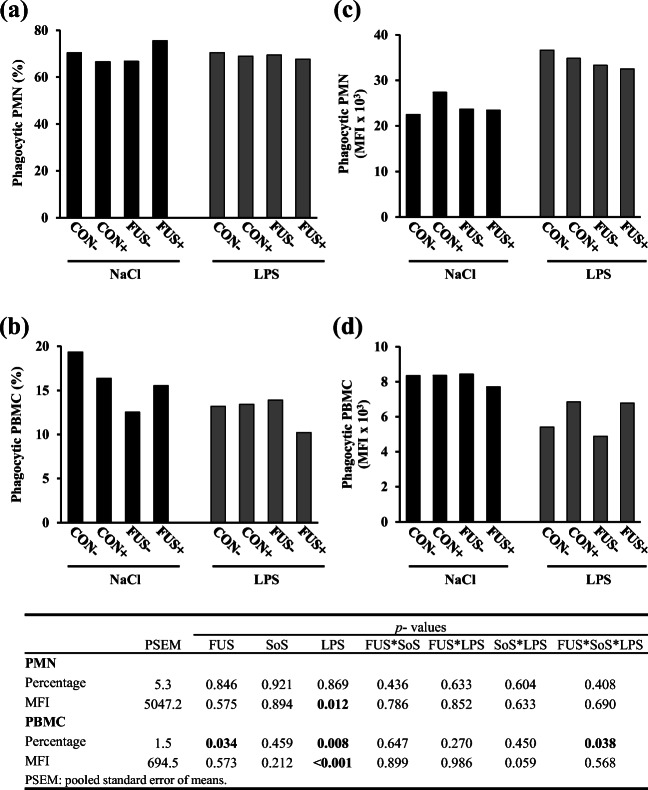
Phagocytic activity was evaluated in PMN (= granulocytes) and PBMC (= monocytes) with the use of FITC-labelled *E. coli* bacteria. The percentage of phagocyting PMN (**a**) and PBMC (**b**) and their respective MFI values (**c**, **d**) are presented as LSMeans (*n* = 5). Piglets received experimental diets for 5 weeks and were subjected to a subsequent acute LPS challenge. Diets contained control (CON) or *Fusarium*-toxin contaminated maize (FUS), wet-conserved with or without 5 g SoS/kg maize sodium sulfite (+/−) and piglets were injected with 7.5 μg LPS/kg BW or 0.9% NaCl

Data on phagocytosis in PMN and PBMC population are represented in Fig. 7. The percentage of phagocytic PMN (Fig. 7a) was not altered by any treatment and remained at its mean value 69.5 ± 5.3%. However, we observed a significant increase in MFI of LPS-challenged pigs (Fig. 7c), indicating a higher phagocytic activity per cell in PMN. The percentage of phagocytic PBMC (Fig. 7b) was significantly altered as indicated by an interaction between factors FUS, SoS treatment, and LPS challenge: in CON-fed groups, LPS challenge decreased percentage of phagocytic cells, independent of SoS presence. However, in FUS-fed groups the LPS effect was dependent on SoS presence: in group FUS−, the percentage of phagocytic cells slightly increased after LPS challenge, whereas in FUS+, the percentage markedly dropped. PBMC of LPS-challenged pigs showed a decrease in MFI (Fig. 7d), indicating a lower phagocytic activity per cell.

## Discussion

Based on the cytotoxic effects of DON and sulfiting agents in general, and the reported interactive effects between SoS treatment of maize and LPS-induced systemic inflammation on the leukocyte count subsets in particular (Tran et al. 2018b), we hypothesized that DON and/or SoS treatment of maize would affect the immune traits with regard to the proportion of lymphocyte subsets as well as cellular function of granulocytes and monocytes.

LPS-induced systemic inflammation is based on a cascade of release of inflammatory mediators, vascular and physiological changes, and a recruitment of immune cells (van Amersfoort et al. 2003) resulting in a redistribution of immune cells between blood, lymph nodes, spleen, other organs and the site of the stimulus. Initial LPS response includes an activation of monocytes and macrophages due to binding simultaneously to the sCD14 (soluble form) in the blood and to the mCD14 (membrane-bound form) (Metkar et al. 2012). LPS-activated macrophages produce intracellular oxygen-free radicals and microbicidal agents and release various inflammatory mediators, which in turn initiate an acute phase reaction resulting in an array of effects on surrounding tissues (van Amersfoort et al. 2003). In agreement with literature, our results revealed a lower percentage of CD14^+^ cells in blood in LPS-challenged pigs. This observation is further substantiated by the LPS-reduced absolute monocyte counts in blood. Furthermore, our previous evaluation on the differential blood counts revealed an interactive effect between FUS maize, SoS treatment, and LPS injection for the monocyte counts with a general decrease due to LPS, a marked lower number in pooled groups FUS− and CON+ compared with control group CON−, and an increase in group FUS+ (Tran et al. 2018b). In the present study, a higher percentage of CD14^+^ cells in both mesenteric lymph node and spleen in pigs fed FUS maize suggests a regulatory effect of DON on the migration of blood monocytes into lymphatic tissues.

As the first response of host defense, the innate immune cells including monocytes and neutrophils activate the phagocytosis and promote the release of oxygen free radicals, also called reactive oxygen species (ROS) in order to kill the invading microbial pathogen (Goodbourn et al. 2000; Fialkow et al. 2007). Although our results showed a significant three-factorial interaction between FUS maize, SoS treatment, and LPS injection for the percentage of phagocytosing PBMC, this effect was seemingly related to the LPS and DON impact with a general alteration in pooled LPS-challenged groups and in pooled FUS groups. Additionally, the significant lower MFI of phagocytosing PBMC in LPS-challenged pigs could reflect the impairment of LPS on the phagocytic activity per cell. In addition to phagocytosis, the release of ROS is responsible for killing the invading microorganism; however, it has been further suggested that an excessive ROS production leads to oxidative stress (Mishra et al. 2014). Besides, LPS-induced oxidative stress has been reported in various studies (Hsu and Wen 2002; Hsu and Liu 2004; Gasparrini et al. 2017). In accordance with the literature, our present results showed an increased proportion in basal ROS-generated PBMC in LPS-challenged pigs. Mitochondria are considered the center of metabolic pathway, a bioenergetic organelle with multiple functions such as energy supply, biosynthesis, and signaling platform for various innate immune signaling pathways. Recently, it has been reported that the mitochondrial respiratory chain in macrophages is adapted for contributing to antibacterial host defense due to ROS signaling to mitochondrial electron transport chain (Garaude et al. 2016). Thus, an increase for ROS generation in PBMC with LPS could reflect the increased metabolic activity and also increased energy demand in monocytes suggesting an impact of LPS on the mitochondrial respiratory chain.

Similar to monocytes/macrophages, the neutrophil granulocytes not only promote phagocytosis but also induce the ROS production in response to LPS (van Amersfoort et al. 2003). The significant effect of LPS on the MFI of phagocytosing granulocytes might be a reflection of a higher phagocytic activity per cell. Our results also revealed that LPS significantly increased the percentage of basal ROS production in granulocytes. Moreover, the significant interaction between SoS treatment and LPS on the MFI of basal ROS formation of PMN would suggest a SoS-modulated LPS effect on this cell type. These observations might be further supported by our previous data on the differential white blood counts, whereby an interactive effect between FUS maize and SoS treatment was observed for the amount of granulocytes with the higher number of granulocytes in the pooled group FUS+ compared with the group FUS− despite a general LPS reduction (Tran et al. 2018b). In a study with human PBMC, Winkler et al. (2006) concluded that SoS suppressed the release of cytokine INF-γ, which is the most important mediator responsible for ROS formation (Karin and Greten 2005; Winkler et al. 2006). Thus, SoS may act as an antioxidant preventing ROS formation due to a reduced INF-γ release, which however was not measured in our study.

The present data revealed that the alterations of T lymphocyte subsets mostly occurred in the peripheral blood samples. In blood, higher proportions of CD4^+^ T cells, the so-called T helper cells, were observed in LPS-treated piglets, confirming an activated state of the immune system (Gerner et al. 2009; Gerner et al. 2015) after the LPS challenge. This interpretation is further supported by the CD4^+^/CD8^+^ cell ratio, which is regarded as critical in maintaining a stable immunological function (Dou et al. 2013). Looking closer, LPS injection induced an increase of CD4^+^/CD8^+^ cell ratio in LPS-challenged piglets, which agreed well with the findings of another study using a similar LPS challenge protocol in rearing piglets (Stelter et al. 2013). With regard to SoS treatment, lower proportions of CD4^+^ T cells in blood were found in pigs receiving SoS treated maize, which resulted in lower CD4^+^/CD8^+^ ratio. Winkler et al. (2006) demonstrated in an in vitro study on human PBMC that the food preservative SoS had a suppressive impact on activated T helper cells of the type Th1. Therefore, the SoS-related reduction of CD4^+^ T cells and a change of CD4^+^/CD8^+^ ratio in our study might be partially attributed to the impact on Th1 cell functionality. Moreover, the release of cytokines is considered the crucial factors for activation of T helper cell types such as IFN-γ, IL-2, IL-12 (Th1) and IL-4, IL-5, IL-9 (Th2) which however were not measured in the present study (Romagnani 2004; Murr et al. 2005; Winkler et al. 2006).

In the literature, the higher percentage of porcine double-positive T cells (CD3^+^CD4^+^CD8^+^) in blood and lymphoid organs as compared with humans has been reported (Zuckermann and Husmann 1996; Waters et al. 1999; Zuckermann 1999). Our results revealed an increase in double-positive T cells in blood when pigs were fed FUS-toxin contaminated maize. Moreover, the significant interaction between FUS maize and LPS challenge for double-positive cells in mesenteric lymph node might reflect a DON-related effect since the percentage of double-positive T cells was only decreased in the pooled group FUS/NaCl. Contrarily, a tendency of an interactive effect between FUS maize and LPS challenge in blood (*p*_FUS*LPS_ = 0.078, Fig. 1) was observed due to an increase in the pooled group FUS/NaCl. Therefore, the higher percentage of double-positive T cells in blood might be related to migrating cells from secondary lymphoid organs such as mesenterial lymph nodes into peripheral blood. The double-positive T cells are considered memory cells and play a role in protective immunity and immune regulation (Zuckermann 1999).

For the double-negative T cells (CD3^+^CD4^−^CD8^−^), similar observations have been made: a decrease of this cell type in spleen, whereas a marked increase in blood in pigs fed FUS maize. It has been suggested that in thymus, the double-negative CD4^−^CD8^−^ precursor cells differentiate into CD4^+^CD8^+^ thymocytes, which upon further differentiation lose either CD4 or CD8, giving rise to the mature CD4^+^ and CD8^+^ single-positive cells (Gerner et al. 2015). In mise, the extrathymic double-negative T cells seemingly relate to defective differentiation. However, analyses of porcine T cells in blood and secondary lymphatic organs revealed several peculiarities compared with humans and rodent species, whereby the presence of double-negative T cells are predominant in spleen (Saalmüller et al. 1987). Moreover, it has been proposed that the double-negative cells are not homogeneous and might also comprise other subpopulations including B cells and monocytes (Arriëns et al. 1998). Thus, the immune-modulating effects of DON on the double-negative cells might be associated with the alteration of other immune cell types. Our data indicate that the significant impact of FUS maize was only evident in double-positive or double-negative T cell subsets. In the frame of our experimental setup, we are unable to address whether double-negative cells lose their epitopes and transform into double-negative cells or differentiate in other subsets (i.e., CD8^+^).

Although the alteration of T cell subpopulation only occasionally occurred in the lymphatic tissues, the present results demonstrated significant three-factorial interaction for CD8^+^ T cells in mesenteric lymph node. It seemed that SoS treatment only of the FUS-contaminated maize group (FUS+/LPS) prevented the LPS-induced increase of CD8^+^ T cell proportion observed in group FUS−/LPS. This observation might be related to the presence of DON sulfonates, both in systemic and local immune system.

It has been suggested that swine showed a higher percentage of CD8^+^ T cells in blood compared with humans (Lunney and Pescovitz 1987; Saalmüller et al. 1989; Lorenzen et al. 2015). The CD8^+^ T cells are known as cytotoxic cells and can attack directly pathogen-infected cells (Gerner et al. 2009). Therefore, it was of interest to examine the CD8^+^ T cell subsets, which can be subdivided into CD8^low^ and CD8^high^ subsets. CD8^high^ subset is considered the cytotoxic T cells due to a strong cytolytic activity (Gerner et al. 2009), whereas CD8^low^ cells might act as T helper–like cells due to the expression of IFN-γ and IL-4, the signature cytokines of type 1 (Th1) and 2 (Th2) T cells (Lunney and Pescovitz 1987). The effect of DON on both subsets was rarely examined and showed no effect on the CD8^high^ subset in pigs fed diet containing 0.5 mg DON (Ferrari et al. 2009). Contrarily, present results suggested that the MFI (giving an indication of the density of CD epitopes) of CD8^high^ cells in pigs fed FUS maize was increased which was paralleled by a slightly higher percentage of CD8^high^ cells, suggesting that dietary exposure to DON might change the cytotoxic T cell function due to increased expression of CD8 receptor on the T cell surface. The DON effect on the immune response is considered to depend on dose, exposure frequency, and timing of functional immune assay (Pestka et al. 2004). Thus, the reason for the different results between studies might be associated with higher dosage of DON, which was about ten-fold higher in our study.

With regard to SoS treatment, our data revealed significant SoS effects on both CD8^low^ and CD8^high^ cells. Studies on the SoS toxicity showed that SoS has a very low level of mammalian toxicity and is free from carcinogenic activity (Nair and Elmore 2003). Once ingested, SoS and other sulfite salts are metabolized to sulfate by the enzyme sulfite oxidase which is excreted and less toxic than sulfite (Dänicke et al. 2012). However, this conversion is determined by the species-specific enzyme activity (Tejnorova 1978) and appears to be incomplete. Moreover, a very low level of sulfite oxidase activity was found in macrophages and neutrophils compared with other cell types such as hepatocytes. Consequently, non-metabolized sulfite might affect these cell types (Beckspeier et al. 1985). It was shown that sulfite did not suppress the cell viability (Winkler et al. 2006). Additionally, an amount of sulfite still existed in blood when pigs were fed SoS-treated maize, although a higher whole sulfuric concentration was not observed in groups compared with control groups (Tran et al. 2018a). Therefore, these present sulfite concentrations might affect immune cells due to the alteration of CD8 epitope expression.

CD21 is only expressed by mature B cells and the proportion of this cell type is regarded as an indicator for B cell development (Axcrona et al. 1996). Present results demonstrated that the CD21^+^ B cell proportion in blood was altered due to an interactive effect between FUS and LPS. This observation is further supported by a similar alteration of lymphocyte counts in blood as published earlier (Tran et al. 2018b): a decrease of lymphocytes in group FUS−/NaCl and CON−/LPS, but an increase of this cell type in group FUS−LPS. In addition, a tendency (*p*_FUSxLPS_ = 0.054) for opposing effects of FUS maize and LPS injection on MFI of CD21^+^cells in blood was also observed with a similar reduction in pooled groups FUS/NaCl and CON/LPS, suggesting a suppressive effect of DON and LPS on the circulating B cells and might reflect the migration activity of this cell type. However, this putative migration was not paralleled by an altered B cell percentage in lymphatic organs. Interestingly, the significant interaction between FUS maize and SoS treatment for the MFI of splenic CD21^+^ B cells could hint at a DON-related effect since the MFI level was higher in group FUS+, which reflects some alterations in the expression of CD21 molecules on the B cell surface in lymphatic organs. The CD21 epitope as an indicator for B cell development interacts with CD2 and forms the CD2/CD21 complex, leading to further differentiation of mature B cells. The latter CD2 expression, however, was not measured in our study.

DON might affect immune traits, especially the immunoglobulin A (IgA) production as a specific effect of DON (Döll and Dänicke 2011; Pestka 2003), whereas DON effects on B cells itself were rarely investigated. In horse, Khol-Parisini et al. (2012) observed no effect of DON on the CD21^+^ B cells when horses were fed highly DON-contaminated oat with 20.2 mg DON/kg oat. Other studies with mice reported that DON has no direct impact on the primary B cells from Peyer’s patches or spleen or in cloned B cells, but rather indirectly due to the influence on the T cell subpopulations and macrophages (Pestka 2003).

In conclusion, the results of this study indicate that alterations in the subpopulations of lymphocytes and monocytes mostly occurred in the peripheral blood and only occasionally in the investigated mesenteric lymph node and spleen tissues. In particular, SoS treatment of maize altered the T-lymphocyte subpopulations in blood with a more pronounced effect on CD8^low^ and CD8^high^ subsets, whereas impact of FUS was more notable on the double-positive T cells in the lymphatic tissues. Furthermore, SoS treatment of maize partially suppressed the magnitude of an LPS effect on cellular function, irrespective of diet. Further studies are needed to elucidate the observed effects, in particular with regard to cell function in the various subsets and tissues.

## Electronic supplementary material

Fig S1 Gating strategy of four T-cell subsets. (PPTX 61 kb)

Fig S2 Gating strategy of B-cells and monocytes. (PPTX 57 kb)

Fig S3 Mean fluorescence intensity (MFI) in total CD4^+^ and CD8^+^ cells in blood, mesenteric lymph node and spleen (LSMeans, n = 5) in piglets receiving experimental diets for 5 weeks and subjected to a subsequent acute LPS challenge. Diets contained control (CON) or *Fusarium*-toxin contaminated maize (FUS), wet-conserved with or without 5 g SoS/kg maize sodium sulfite (+/-) and piglets were injected with 7.5 μg LPS/kg BW or 0.9% NaCl. (PPTX 49 kb)

## Abbreviations

CON: Control maize
DON: Deoxynivalenol
FUS: *Fusarium*–toxin contaminated maize
LOD: Level of detection
LPS: Lipopolysaccharide
MFI: Mean fluorescence intensity
PBMC: Peripheral blood mononuclear cells
PMN: Polymorphonuclear cells
ROS: Reactive oxygen species
SoS: Sodium sulfite
