# Supplementary material for: Sodium sulfite (SoS) as decontamination strategy for Fusarium-toxin contaminated maize and its impact on immunological traits in pigs challenged with lipopolysaccharide (LPS)
Source: Mycotoxin Res. 2020 Sep 9;36(4):429–42. doi: 10.1007/s12550-020-00403-x (PMC7536171; doi:10.1007/s12550-020-00403-x)
Supplement: Supplementary file 1 — Gating strategy of four T-cell subsets. (PPTX 61 kb) [file 12550_2020_403_MOESM1_ESM.pptx]

## Slide 1
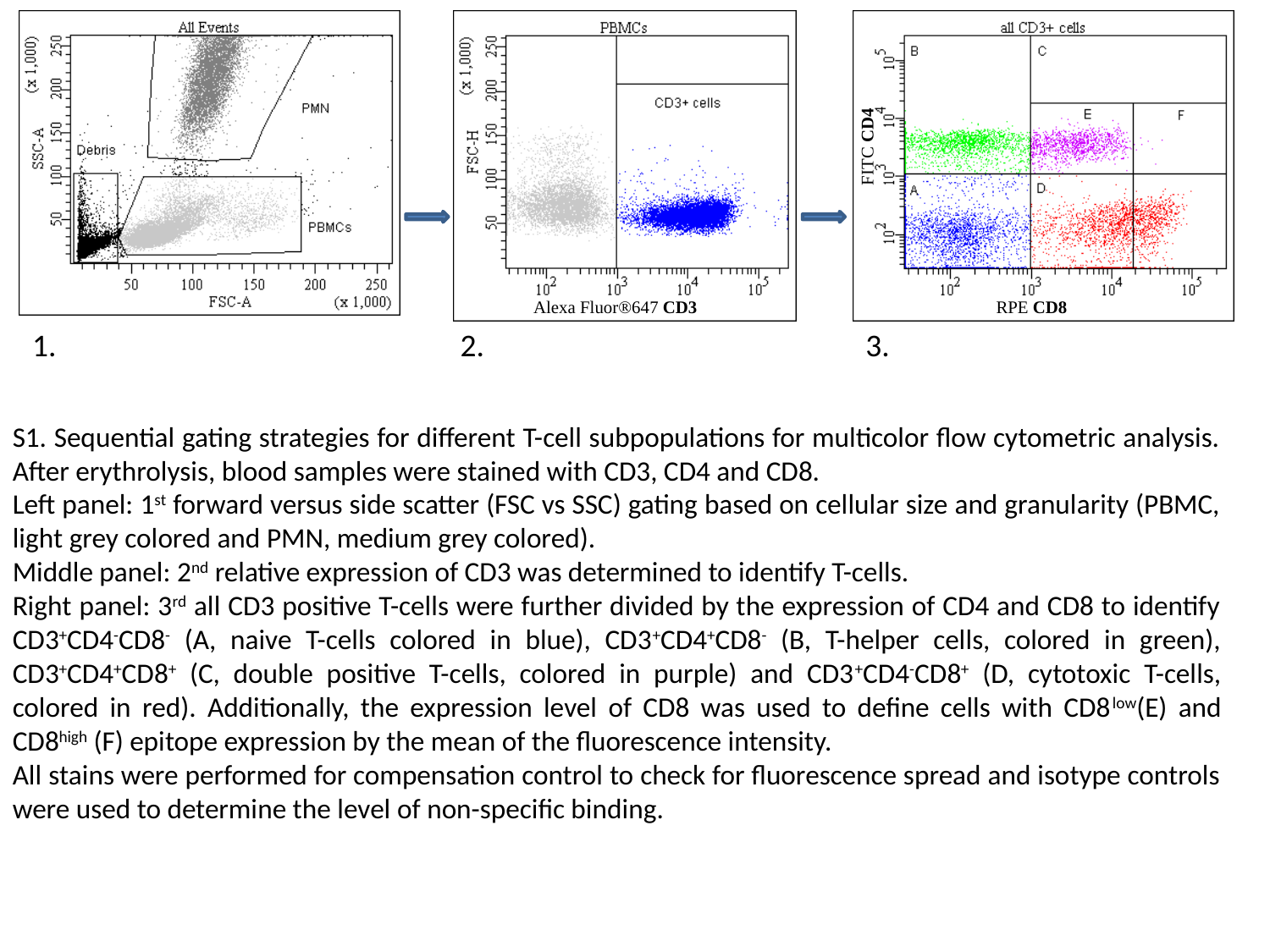

FITC CD4
RPE CD8
Alexa Fluor®647 CD3
1.
2.
3.
S1. Sequential gating strategies for different T-cell subpopulations for multicolor flow cytometric analysis. After erythrolysis, blood samples were stained with CD3, CD4 and CD8.
Left panel: 1st forward versus side scatter (FSC vs SSC) gating based on cellular size and granularity (PBMC, light grey colored and PMN, medium grey colored).
Middle panel: 2nd relative expression of CD3 was determined to identify T-cells.
Right panel: 3rd all CD3 positive T-cells were further divided by the expression of CD4 and CD8 to identify CD3+CD4-CD8- (A, naive T-cells colored in blue), CD3+CD4+CD8- (B, T-helper cells, colored in green), CD3+CD4+CD8+ (C, double positive T-cells, colored in purple) and CD3+CD4-CD8+ (D, cytotoxic T-cells, colored in red). Additionally, the expression level of CD8 was used to define cells with CD8low(E) and CD8high (F) epitope expression by the mean of the fluorescence intensity.
All stains were performed for compensation control to check for fluorescence spread and isotype controls were used to determine the level of non-specific binding.
