# Supplementary material for: Sodium sulfite (SoS) as decontamination strategy for Fusarium-toxin contaminated maize and its impact on immunological traits in pigs challenged with lipopolysaccharide (LPS)
Source: Mycotoxin Res. 2020 Sep 9;36(4):429–42. doi: 10.1007/s12550-020-00403-x (PMC7536171; doi:10.1007/s12550-020-00403-x)
Supplement: Supplementary file 2 — Gating strategy of B-cells and monocytes. (PPTX 57 kb) [file 12550_2020_403_MOESM2_ESM.pptx]

## Slide 1
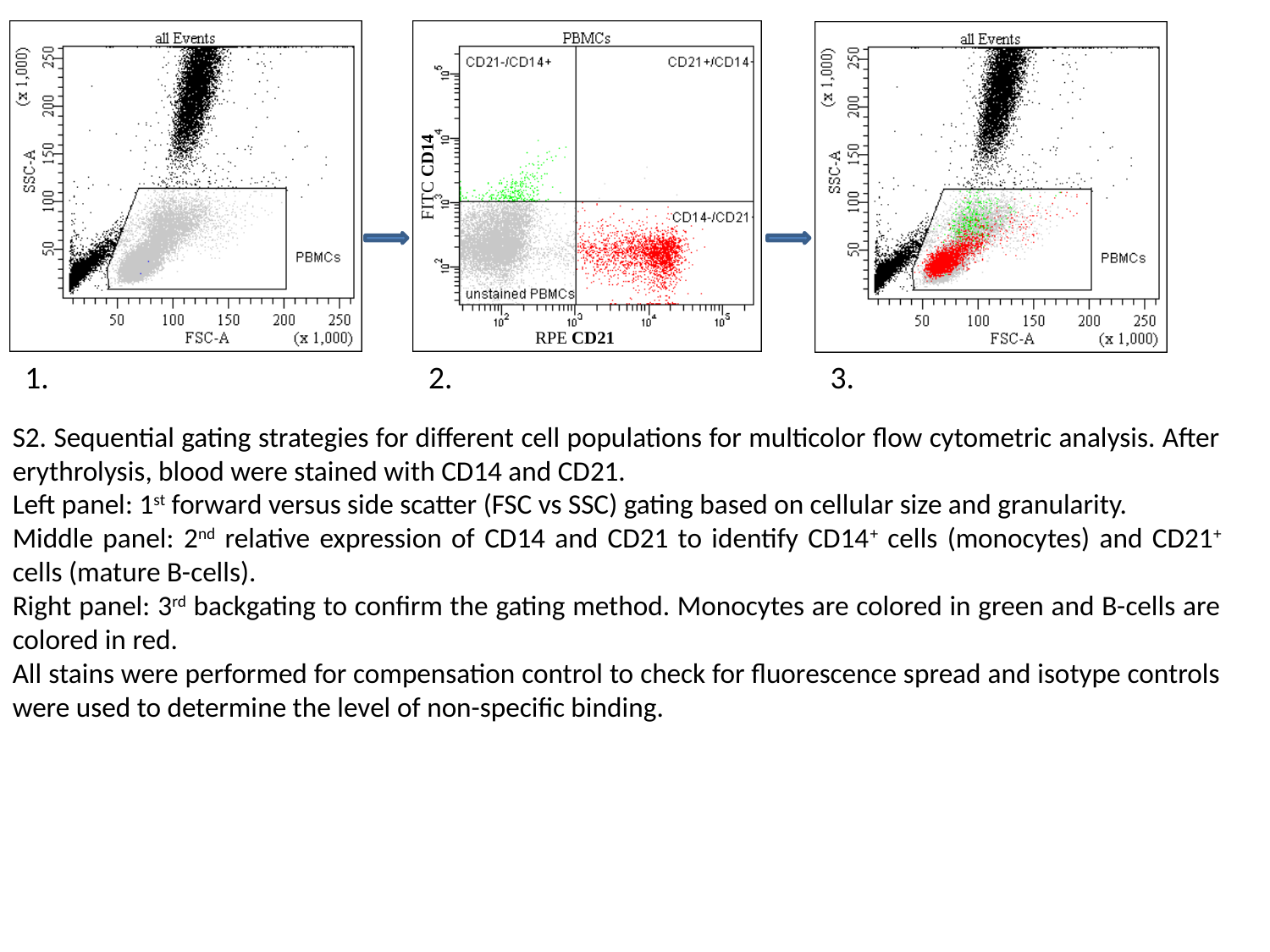

FITC CD14
RPE CD21
1.
2.
3.
S2. Sequential gating strategies for different cell populations for multicolor flow cytometric analysis. After erythrolysis, blood were stained with CD14 and CD21.
Left panel: 1st forward versus side scatter (FSC vs SSC) gating based on cellular size and granularity.
Middle panel: 2nd relative expression of CD14 and CD21 to identify CD14+ cells (monocytes) and CD21+ cells (mature B-cells).
Right panel: 3rd backgating to confirm the gating method. Monocytes are colored in green and B-cells are colored in red.
All stains were performed for compensation control to check for fluorescence spread and isotype controls were used to determine the level of non-specific binding.
