# Supplementary material for: Sodium sulfite (SoS) as decontamination strategy for Fusarium-toxin contaminated maize and its impact on immunological traits in pigs challenged with lipopolysaccharide (LPS)
Source: Mycotoxin Res. 2020 Sep 9;36(4):429–42. doi: 10.1007/s12550-020-00403-x (PMC7536171; doi:10.1007/s12550-020-00403-x)
Supplement: Supplementary file 3 — Mean fluorescence intensity (MFI) in total CD4+ and CD8+ cells in blood, mesenteric lymph node and spleen (LSMeans, n = 5) in piglets receiving experimental diets for 5 weeks and subjected to a subsequent acute LPS challenge. Diets contained control (CON) or Fusarium-toxin contaminated maize (FUS), wet-conserved with or without 5 g SoS/kg maize sodium sulfite (+/-) and piglets were injected with 7.5 μg LPS/kg BW or 0.9% NaCl. (PPTX 49 kb) [file 12550_2020_403_MOESM3_ESM.pptx]

## Slide 1
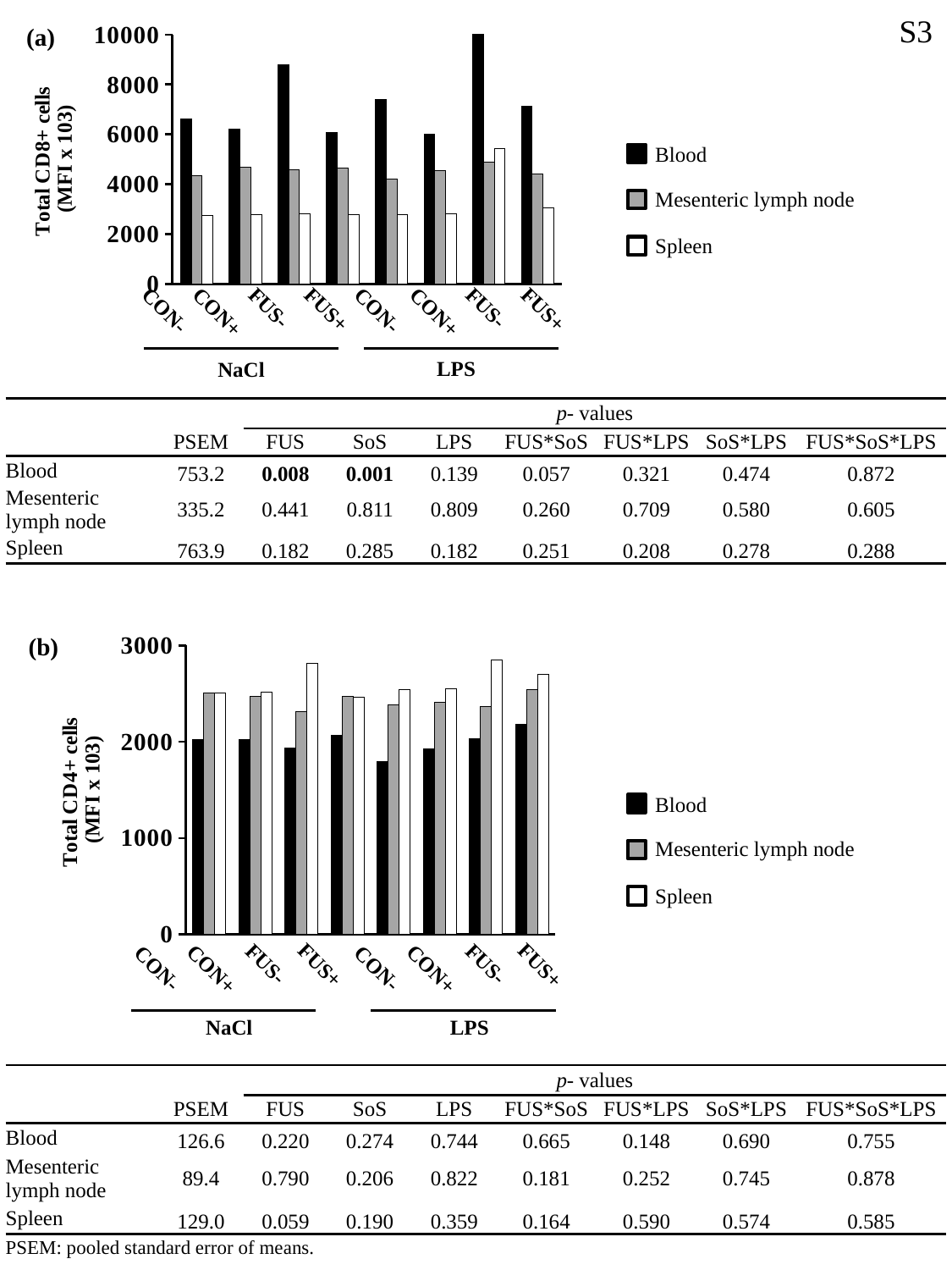

S3
### Chart
| Category | blood | mlymph | spleen |
|---|---|---|---|
| CON-/NaCl | 6623.8 | 4344.2 | 2732.3 |
| CON+/NaCl | 6216.4 | 4687.2 | 2790.4 |
| FUS-/NaCl | 8777.0 | 4591.8 | 2824.8 |
| FUS+/NaCl | 6060.0 | 4634.0 | 2785.0 |
| CON-/LPS | 7380.0 | 4203.8 | 2788.9 |
| CON+/LPS | 6012.6 | 4529.2 | 2820.3 |
| FUS-/LPS | 10449.4 | 4884.0 | 5435.3 |
| FUS+/LPS | 7123.2 | 4406.0 | 3036.6 |CON-
FUS-
CON-
FUS-
CON+
FUS+
CON+
FUS+
LPS
NaCl
(a)
Blood
Mesenteric lymph node
Spleen
| | | | p- values | | | | | | |
| --- | --- | --- | --- | --- | --- | --- | --- | --- | --- |
| | | PSEM | FUS | SoS | LPS | FUS\*SoS | FUS\*LPS | SoS\*LPS | FUS\*SoS\*LPS |
| Blood | | 753.2 | 0.008 | 0.001 | 0.139 | 0.057 | 0.321 | 0.474 | 0.872 |
| Mesenteric lymph node | | 335.2 | 0.441 | 0.811 | 0.809 | 0.260 | 0.709 | 0.580 | 0.605 |
| Spleen | | 763.9 | 0.182 | 0.285 | 0.182 | 0.251 | 0.208 | 0.278 | 0.288 |
| | | | | | | | | | |
### Chart
| Category | blood | mlymph | spleen |
|---|---|---|---|
| CON-/NaCl | 2024.4 | 2503.8 | 2509.0 |
| CON+/NaCl | 2020.8 | 2468.6 | 2515.5 |
| FUS-/NaCl | 1936.6 | 2316.8 | 2818.7 |
| FUS+/NaCl | 2067.6 | 2474.0 | 2464.4 |
| CON-/LPS | 1798.0 | 2385.2 | 2542.8 |
| CON+/LPS | 1922.8 | 2411.0 | 2552.4 |
| FUS-/LPS | 2031.8 | 2365.4 | 2851.1 |
| FUS+/LPS | 2178.6 | 2544.4 | 2701.2 |(b)
FUS-
FUS-
FUS+
FUS+
CON-
CON-
CON+
CON+
NaCl
LPS
Blood
Mesenteric lymph node
Spleen
| | | | p- values | | | | | | |
| --- | --- | --- | --- | --- | --- | --- | --- | --- | --- |
| | | PSEM | FUS | SoS | LPS | FUS\*SoS | FUS\*LPS | SoS\*LPS | FUS\*SoS\*LPS |
| Blood | | 126.6 | 0.220 | 0.274 | 0.744 | 0.665 | 0.148 | 0.690 | 0.755 |
| Mesenteric lymph node | | 89.4 | 0.790 | 0.206 | 0.822 | 0.181 | 0.252 | 0.745 | 0.878 |
| Spleen | | 129.0 | 0.059 | 0.190 | 0.359 | 0.164 | 0.590 | 0.574 | 0.585 |
| PSEM: pooled standard error of means. | | | | | | | | | |
